# *Insilico* analysis of hypothetical proteins unveils putative metabolic pathways and essential genes in *Leishmania donovani*

**DOI:** 10.3389/fgene.2014.00291

**Published:** 2014-08-26

**Authors:** Nithin Ravooru, Sandesh Ganji, Nitish Sathyanarayanan, Holenarsipur G. Nagendra

**Affiliations:** ^1^Department of Biotechnology, Sir Mokshagundam Visvesvaraya Institute of TechnologyBangalore, India; ^2^The National Centre for Biological Sciences, Tata Institute of Fundamental ResearchBangalore, India

**Keywords:** *Leishmania donovani*, hypothetical proteins, ubiquinone biosynthesis, seleno-cysteine metabolism, fatty acid elongation, drug targets

## Abstract

Leishmaniasis is a parasitic disease caused by the protozoan Leishmania, which is active in two broad forms namely, *Visceral Leishmaniasis* (VL or Kala Azar) and *Cutaneous Leishmaniasis* (CL). The disease is most prevalent in the tropical regions and poses a threat to over 70 countries across the globe. About 200 million people are estimated to be at risk of developing VL in the Indian subcontinent, and this refers to around 67% of the global VL disease burden. The Indian state of Bihar alone accounts for 50% of the total VL cases. While no vaccination exists, several pentavalent antimonials and drugs like Paromomycin, Amphotericin, Miltefosine etc. are used in the treatment of Leishmaniasis. However, due to their low efficacies and the resistance developed by the bug to these medications, there is an urgent need to look into newer species specific targets. The proteome information available suggests that among the 7960 proteins in *Leishmania donavani*, a staggering 65% remains classified as a hypothetical uncharacterized set. In this background, we have attempted to assign probable functions to these hypothetical sequences present in this parasite, to explore their plausible roles as druggable receptors. Thus, putative functions have been defined to 105 hypothetical proteins, which exhibited a GO term correlation and PFAM domain coverage of more than 50% over the query sequence length. Of these, 27 sequences were found to be associated with a reference pathway in KEGG as well. Further, using homology approaches, four pathways viz., Ubiquinone biosynthesis, Fatty acid elongation in Mitochondria, Fatty Acid Elongation in ER and Seleno-cysteine Metabolism have been reconstructed. In addition, 7 new putative essential genes have been mined with the help of Eukaryotic Database of Essential Genes (DEG). All these information related to pathways and essential genes indeed show promise for exploiting the select molecules as potential therapeutic targets.

## Introduction

Leishmaniasis is a parasitic disease caused by the protozoan belonging to the genus Leishmania and is transmitted by the vector phlebotomine or sand fly (Alvar et al., 2013). The disease is active in two broad forms namely Visceral Leishmaniasis (VL or Kala Azar) and Cutaneous Leishmaniasis (CL) (Croft et al., 2006). Visceral Leishmaniasis is the more severe form of the disease, characterized by anemia, splenohepatomegaly, depressed immune response and several secondary infections finally leading to death (Alvar et al., 2013). VL is primarily caused by the protozoan *Leishmania donovani* in the South Asia and African regions and by *Leishmania infantum* in the central and South American regions (Croft and Olliaro, 2011).

The disease is most prevalent in the tropical regions and poses a threat to over 70 countries across the globe. Approximately, there are 0.7–1.2 million cases of VL and CL respectively, recorded each year and about 20,000–40,000 Leishmaniasis deaths occur per year. The 10 countries with the highest estimated cases namely Afghanistan, Algeria, Colombia, Brazil, Iran, Syria, Ethiopia, North Sudan, Costa Rica and Peru, together account for 70–75% of global estimated CL incidence (Alvar et al., 2012). In the Indian subcontinent, about 200 million people are estimated to be at risk of developing VL and this area harbors an estimated 67% of the global VL disease burden. The north Indian state of Bihar alone has captured almost 50% of the total cases in the Asian region (Bhunia et al., 2013).

Several pentavalent antimonials and drugs like Amphotericin and Paromomycin are currently available as intramuscular injections, while Miltefosine is used as an oral drug, for the treatment of Leishmaniasis. Vector control measures and the first line of drugs have proved incapable of suppressing the disease, especially in India where two thirds of the patients did not respond to these pentavalent antimonials (Lira et al., 1999; Croft et al., 2006; Sundar et al., 2009). The medications are not satisfactory mainly due to their toxicity effects, drug resistance due to their long half-life and the costs associated with the treatment (Desjeux, 2004; Monzote, 2009; Singh et al., 2012). Thus, there is an immense & immediate requirement to look at species specific drug targets to tackle this pathogen (Guerin et al., 2002). The proteome information available suggests that amongst the 7960 protein sequences, a staggering 65% of it remains to be annotated with clarity.

Hence, as a step toward characterization of these hypothetical sequences as plausible drug targets, computational approaches have been employed toward analysing these molecules. Literature suggests that several *insilico* approaches have been adopted in order to assign functional information for such hypothetical sequences in various organisms. More than half of the uncharacterized proteins in *M. tuberculosis* are functionally correlated via computational approaches (Doerks et al., 2012). *Insilico* analysis of hypothetical proteins present in human fetal brain has been predicted to contain many sequences which function in DNA-protein binding and ligase activity (Sharma et al., 2013). Also, *insilico* characterization of hypothetical proteins in *Plasmodium falciparum* suggests that several sequences can be considered as biomarkers in Malaria (Oladele et al., 2011). Recent studies on hypothetical proteins in Trypanosomatids have predicted protein-protein interactions on a genome scale, which could be used to explore new potential drug targets (Rezende et al., 2012). In another recent study in *Trypanosoma cruzi*, attempts have been made to computationally annotate the hypothetical membrane proteins in order to identify putative drug targets (Silber and Pereira, 2012). However, there has been no comprehensive study on the hypothetical protein dataset in *Leishmania donovani* hence; a detailed investigation has been attempted.

## Materials and methods

### Databases employed

Hypothetical sequences were retrieved from UNIPROT-KB (Release 2014_02) (The UniProt Consortium, 2013). KEGG (Release 69.0) database was used for assigning pathway information (Kanehisa et al., 2014). Eukaryote specific Database of Essential Genes (DEG) (Version 10.0) (Luo et al., 2013) was used to search for putative essential genes within *Leishmania donovani*.

### Tools for functional annotation

HMMscan, both web version and standalone (HMMER 3.1b1—Finn et al., 2011) and Batch CDD search (Marchler-Bauer et al., 2011) were used to assign PFAM domain information to the query sequences. Blast2GO (Conesa et al., 2005; Götz et al., 2008) was used to assign functional Gene Ontology (GO) terms (Ashburner et al., 2000) to the protein sequences. KEGG Automatic Annotation Server (KAAS) (Moriya et al., 2007) was used to predict the pathway associations of the protein sequences. String DB (version 9.1) (Franceschini et al., 2013) was used to identify and analyse the COG (cluster of Orthologous groups) (Tatusov et al., 1997) networks between protein families.

### Tools for sequence search and phylogeny

Sequence search algorithm, BLAST was used for identification of homologs where ever necessary. Jackhmmer (HMMER 3.1b1) standalone version was used to search homologs against the Eukaryote DEG database. MEGA 5.0 (Tamura et al., 2011) was used for building phylogenetic tree where Maximum likelihood approach was used. Fig-tree (version 1.4) was used to visualize the phylogenetic tree.

### Sequence analysis

**Figure 1:**
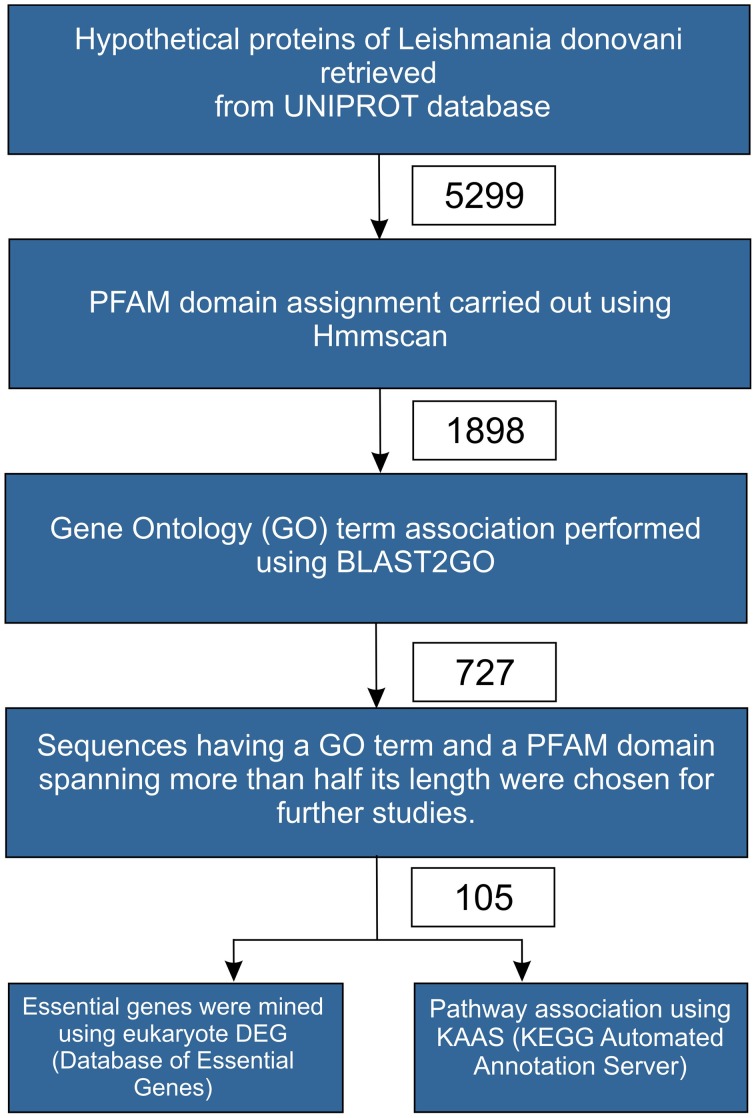
**Protocol used in sequence processing**.

The protocol used in this study is depicted in Figure 1, as a flowchart. 5299 hypothetical protein sequences belonging to *Leishmania donovani* were retrieved from Uniprot. These sequences were analyzed for domain information using HMMscan with an *e*-value of 10^−3^ against the PFAM database (version 27.0) (Finn et al., 2013). We obtained 1898 sequences, which had at least one PFAM domain associated with the query. Further, these 1898 sequences were analyzed for possible GO term associations using Blast2GO tool. The sequences were queried against Swissprot database at an *e*-value of 10^−10^, which resulted in 727 sequences being associated with one or more GO terms. Of the 727 sequences, 105 sequences had a PFAM domain covering more than 50% of the query's length. Hence, these 105 sequences which were outcome of the two filters viz., GO term association and PFAM domain coverage formed the final dataset used for a detailed analysis. The sequences that did not clear the threshold parameters at various filter, may have a higher likelihood of being false positives, and this may warrant a separate in depth analysis of the same.

## Results and discussions

In the present work, we have integrated the available sequences and functional information from various resources to assign putative function to hypothetical proteins. Of the total proteome of 7960 sequences, 5299 proteins in this pathogen are termed hypothetical/uncharacterized, which amounts to a monumental 65% of the proteome that needs to be characterized with structural and functional information. Thus, under the current study 105 sequences have been putatively annotated using sequence/domain information from PFAM, functional information from GO, pathway information from KEGG and essential gene information from DEG.

**Figure 2:**
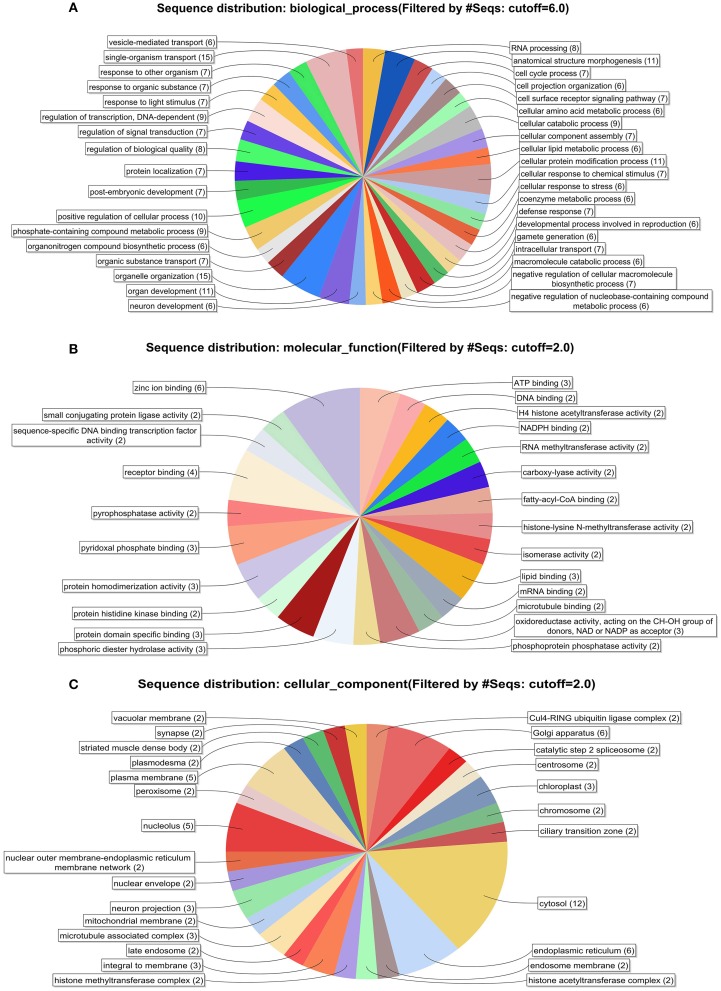
**(A)** Distribution of biological process of the 105 sequences. **(B)** Distribution of molecular function of the 105 sequences. **(C)** Distribution of cellular component of the 105 sequences.

All the information related to GO terms, Interproscan domain associations, homolog's picked during the BLAST step in the BLAST2GO analysis of the 105 sequences, are presented in the Supplementary Table 1 (as an Excel file). Similarly, Supplementary Figure 1 indicates the sequence similarity distribution among the BLAST hits obtained in the first step of Blast2Go analysis. No BLAST hits were present with less than 30% sequence similarity with respect to the query, indicating a good homology with the query. Supplementary Figure 2 shows the *E*-value distribution of the hits from the BLAST step. It is evident from the plot that only the hits with a significant *E*-value were considered for further analysis. Figures 2A–C show combined graphs depicting the biological processes, molecular functions and cellular components of the 105 sequences respectively. Presence of important class of proteins such as Zinc binding, Receptor binding, DNA binding etc., has been highlighted through GO annotation. This information can be extended to investigate the functional roles of these less characterized molecules in greater detail. Further, KEGG Automatic Annotation Server (KAAS) was used to predict Pathway associations for the 105 sequences that have an associated GO term and a domain spanning more than half of its length. KAAS was performed using the Best bidirectional Hit (BBH) method which resulted in 27 sequences associated with 33 KEGG pathways. The complete list of pathways is given in Supplementary Table 2, which provides crucial information related to basic metabolic actions within the protozoa. The 105 sequences when queried against the Eukaryote Database of Essential Genes (DEG), using jackhammer with an *e*-value of 10^−20^, resulted in 26 sequences that had 93 hits from eukaryote DEG.

Here, attempts have been made to reconstruct 4 pathways, viz., Ubiquinone biosynthesis, Fatty acid elongation in Mitochondria, Fatty acid elongation in ER and Seleno-cysteine Metabolism. Sequence homology information is derived from String DB, COG and sequence search tool such as BLAST. Reference pathway information was obtained either from KEGG or MetaCyc databases.

### Homology based pathway reconstruction

**Figure 3:**
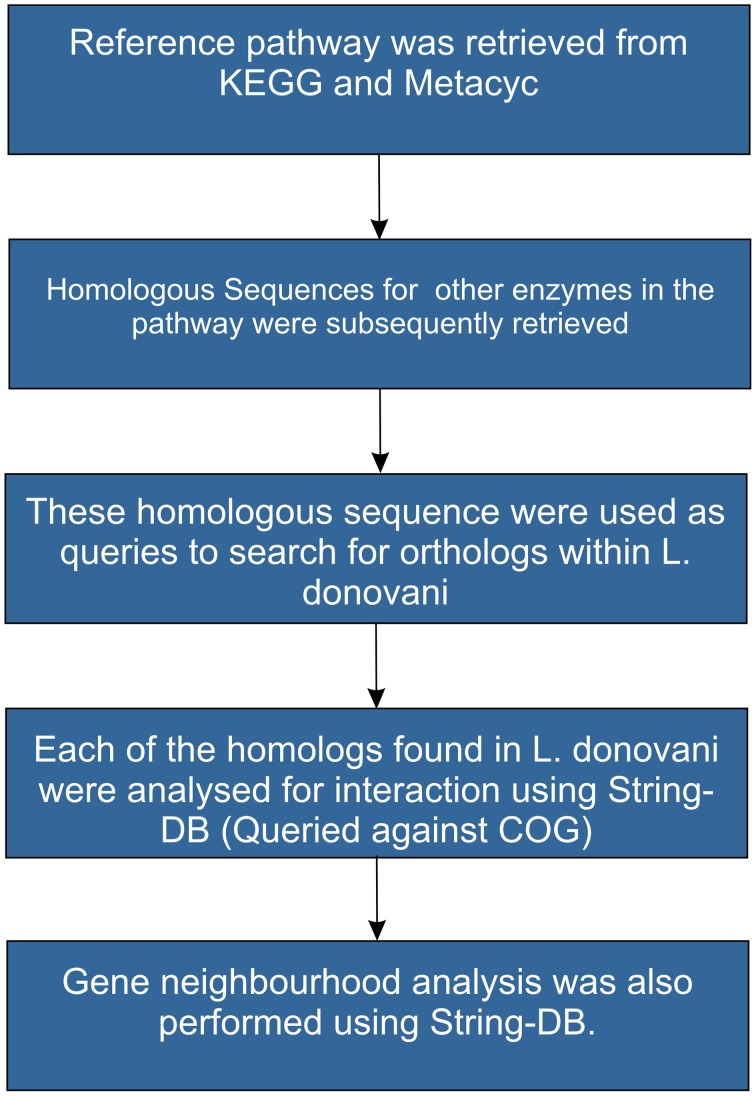
**Protocol depicting steps in homology based pathway reconstruction**.

The protocol involved in homology based pathway reconstruction is depicted in Figure 3. Complete sequence information of the pathways was retrieved from MetaCyc (Caspi et al., 2011). Protein sequence catalyzing each step in the reference pathway was used as a query to search for homologs in *Leishmania donovani* (taxid: 5561) using BlastP at an *e*-value of 10^−3^. Hits which had a coverage of greater than 70% and an identity of >30% were considered as true positives. Further, homologs found within *Leishmania donovani* were used as query to understand the conservation of gene neighborhood using String DB. In a recent study, Doerks et al. (2012) has demonstrated the use of gene neighborhood approach to mine a putative cell envelope biogenesis operon in *Mtb.* Such gene neighborhood, co-occurrence patterns and conservation of proteins in a pathway across evolutionary space signifies the importance of the pathway analysis.

### Case study 1: ubiquinone biosynthesis pathway

In studies involving *Plasmodium* parasites, arrested oocyte maturation is seen in the lack of NADH-Ubiquinone oxidoreductase, which is a part of the electron transport chain (Boysen and Matuschewski, 2011). This shows the importance of ubiquinone and its role within the plasmodium parasite. Subtractive genomic studies in *Mycobacterium tuberculosis* have revealed the possibility of Ubiquinone biosynthesis pathway as targets in multidrug variants of the bacteria (Anishetty et al., 2005).

**Figure 4:**
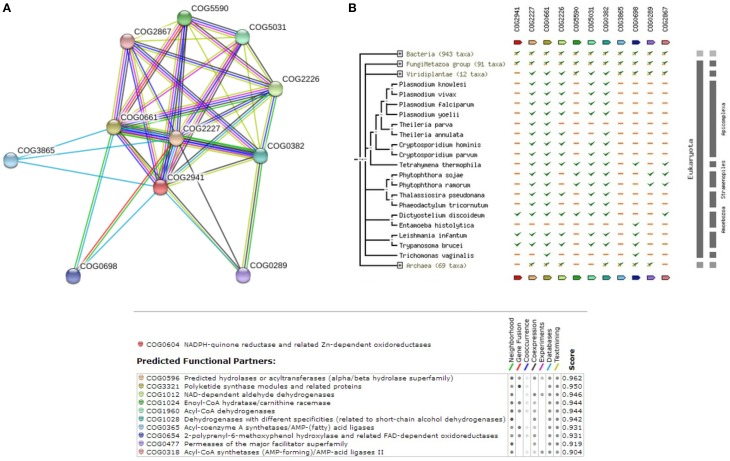
**(A)** COG network for members of Ubiquinone biosynthesis Pathway. **(B)** Phylogenetic profile of the members of Ubiquinone biosynthesis Pathway.

Hence, considering the possibility of ubiquinone biosynthesis pathway as a potential drug target, we explored to reconstruct the pathway in *Leishmania donovani* as well. The complete list of enzymes that are involved in ubiquinone biosynthesis within *Leishmania donovani* is also illustrated in Supplementary Table 3. Supplementary Figure 3 shows the reference pathway and steps involved in ubiquinone biosynthesis. Figure 4A depicts the String-DB interaction of E9BL43 as queried against COG, while Figure 4B illustrates the Phylogenetic profile of query and other members of COG. As can be appreciated from Figure 4B, interaction of 5 out of 7 enzymes [E9BL43 (COG2941), E9BJL4 (COG0382), E9BSV2 (COG2227), E9BAB0 (COG0661), E9BUR6 (COG2226)], catalyzing various reactions within the pathway remain conserved in the genus Leishmania. Additionally, the enzymes in the Ubiquinone biosynthesis pathway are well conserved in other species of Leishmania (sharing >90% similarity and identity) as shown in Supplementary Table 3. Furthermore, it is seen that of the 7 enzymes established in the pathway, 4 are termed uncharacterized (E9BLP8, E9B8Y8, E9BAB0, E9BL43) in UNIPROT. The query sequence E9BL43, which is among the dataset of 105 sequences, is closely related to coq7, which in turn is shown to be closely interacting with structural components like coq4 and coq9 and the functional components like coq5 and coq6 in *Saccharomyces cerevisiae* (Hsieh et al., 2007). This highlights the role of coq7 (E9BL43) in the COQ (Coenzyme Q) biosynthesis pathway and its interactions with other members as COQ is an essential cofactor in mitochondrial respiration.

### Case study 2: fatty acid elongation (in mitochondria) pathway

Over the years, chemotherapy has been the principle method employed in order to control the disease along with the pentavalent antimonials like Paramomycin, which are rather expensive and toxic. Miltefosine [hexadecylphosphocholine (HePC)] was the first drug to be approved for oral administration against the antimony-resistant cases and cutaneous leishmaniasis. Studies have exhibited classical modifications in the lipid composition in membranes of *Leishmania donovani* promastigotes, that are resistant to Miltefosine (Rakotomanga et al., 2005). The study also suggests that the variations in the lipid composition of the membranes (fatty acid composition and length of alkyl chains) in Miltefosine resistant *L. donovani* promastigotes, with that of its wild-type counterparts, could be helpful in identifying biochemical targets which undergo alterations due to drug resistance processes.

**Figure 5:**
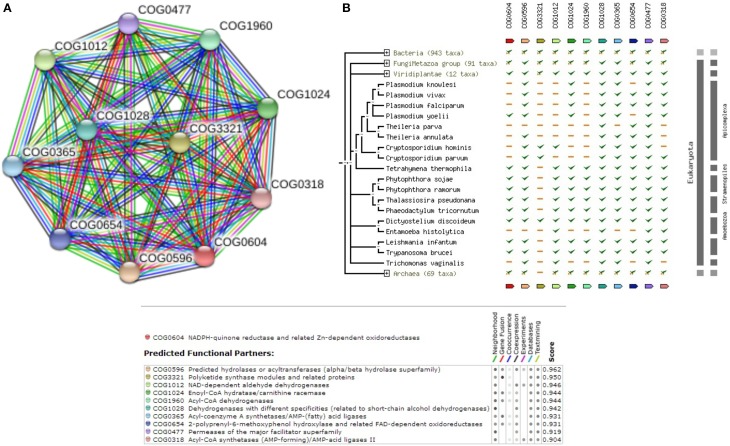
**(A)** COG network display for members of Fatty Acid Elongation (Mitochondria) Pathway. **(B)** Phylogenetic profile of the members of Fatty Acid Elongation (Mitochondria) Pathway.

Hence, understanding the sequence level information of such crucial metabolic pathways related to fatty acid elongation (Mitochondria and endoplasmic reticulum), would aid in better appreciation of Miltefosine induced drug resistance. Supplementary Table 4 shows the complete list of enzymes that are involved in Fatty acid elongation pathway (Mitochondria) within *Leishmania donovani.* The query E9B7Z4 is found to be closely associated to enzyme of the class Trans-2-enoyl-CoA reductase (1.3.1.38). Supplementary Table 4 also shows that the enzymes in the pathway are well conserved in other species of Leishmania (with >90% coverage and similarity). Supplementary Figure 4 shows the reference pathway obtained from KEGG and steps involved in Fatty Acid elongation. Figure 5A shows the String-DB interaction of E9B7Z4 as queried against COG; while Figure 5B shows the phylogenetic profile of query and other members of COG.

### Case study 3: fatty acid elongation (in endoplasmic reticulum) pathway

**Figure 6:**
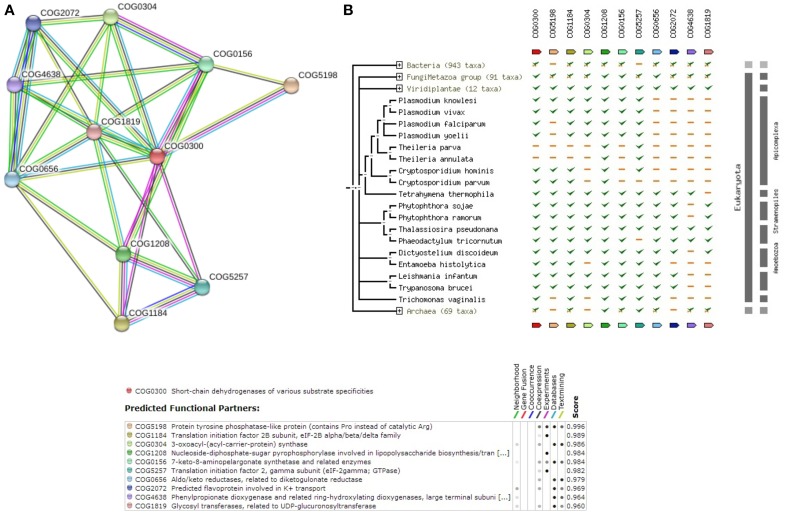
**(A)** COG network display for members of Fatty Acid Elongation (in ER) Pathway. **(B)** Phylogenetic profile of the members of Fatty Acid Elongation (in ER) Pathway.

Fatty acid elongation can occur at Endoplasmic Reticulum (ER) apart from Mitochondria. Since studies have not determined the cellular location of Miltefosine induced drug resistance, it is also important to understand the sequence level information of Fatty acid elongation pathway in ER. The query E9BQF5, which is part of the 105 sequence dataset, is closely related to Ketoacyl-coA-reductase (1.1.1.330). Supplementary Table 5 shows that the enzymes in the pathway are well conserved with other species of Leishmania (with >90% coverage and similarity). Supplementary Figure 5 depicts the reference pathway obtained from KEGG and the steps involved in the pathway. Figure 6A exhibits the String-DB interaction of E9BQF5 as queried against COG while, Figure 6B refers to the phylogenetic profile of the query and other members of COG.

### Case study 4: seleno-cysteine metabolism pathway

Selenoproteins play a wide range of roles in metabolism and oxidative stress defense in many organisms. Seleno-cysteine is synthesized by reaction of seleno-phosphate with a serine charged *t*RNA. The unique reactivity of selenocysteine and the specialized machinery required for selenoprotein synthesis, make selenoproteins an attractive target for antimicrobial development (Jackson-Rosario and Self, 2010). Recently, selenoproteins have been identified in a number of parasitic organisms including trypanosomes and platyhelminths (Lobanov et al., 2006; Bonilla et al., 2008). In addition, selenoproteins in *Plasmodium falciparum* have been suggested as possible targets for therapeutic development (Jackson-Rosario and Self, 2010). Studies have shown that Trypanosoma and Leishmania are sensitive to auranofin, a potent selenoprotein inhibitor; however, the probable drug mechanism is not related to selenoproteins in kinetoplastids. Latest studies have also shown that Selenium supplementation decreases the parasitemia of various Trypanosome infections and reduces important parameters associated with diseases such as anemia and parasite-induced organ damage (Da Silva et al., 2014). It is, thus, interesting to understand the sequence level information of the proteins involved in Seleno-cysteine biosynthesis in *Leishmania donovani*.

**Figure 7:**
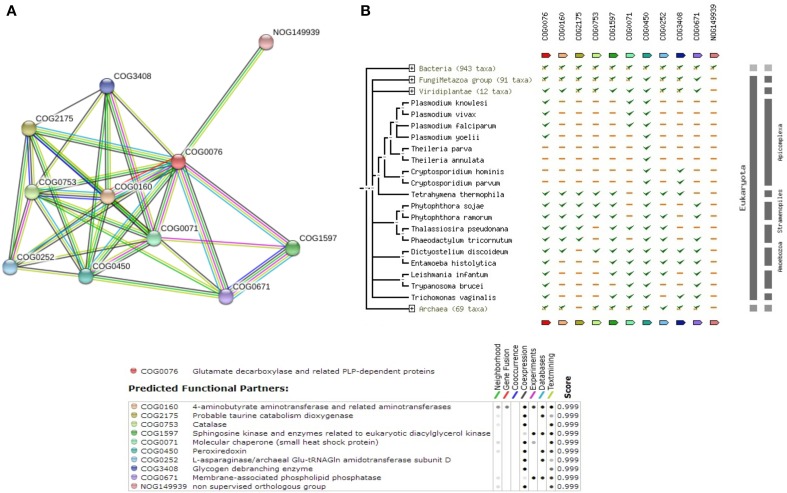
**(A)** COG network display for members of Seleno-cysteine metabolism Pathway. **(B)** Phylogenetic profile of the members of Seleno-cysteine metabolism Pathway.

The query E9B9Y6, which is part of the 105 sequence dataset, is closely related to O-phosphoseryl-tRNA:selenocysteinyl-tRNA synthase (2.9.1.2) which is involved in the synthesis of Selenocysteine. Supplementary Table 6 shows that the enzymes in the pathway are well conserved in other species of Leishmania (with >90% coverage and similarity). Supplementary Figure 6 shows the reference pathway obtained from Metacyc and steps involved in the pathway. Figure 7A depicts the String-DB interaction of E9BQF5 as queried against COG while; Figure 7B illustrates the phylogenetic profile of the query and other members of COG.

### DEG analysis of the 105 sequences

**Figure 8:**
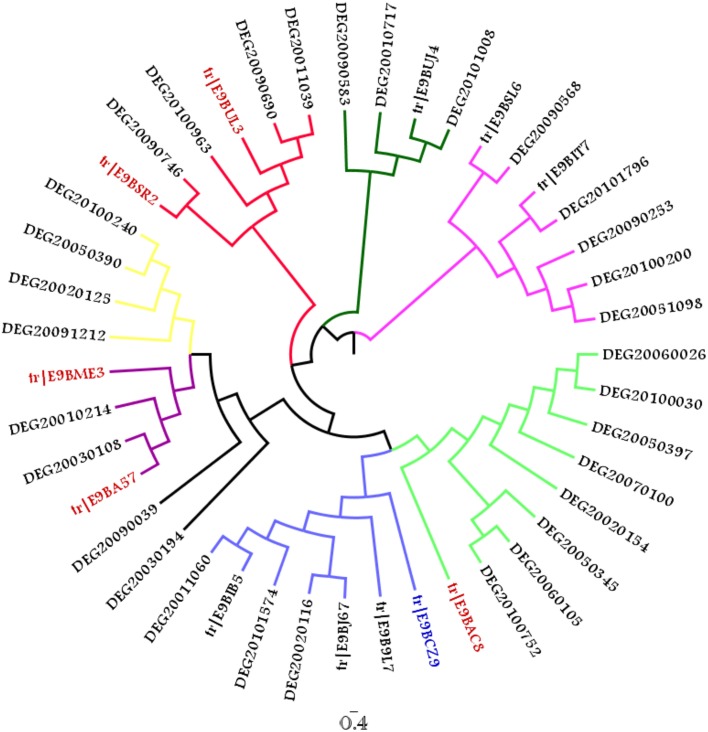
**Phylogenetic tree comprising 12 query associated to 32 DEG hits (The taxa colored in Red correspond to sequences belonging to WD40 superfamily while E9BCZ9, colored in blue is closely related to Nep1, a methyltranferase involved in ribosomal biogenesis)**.

In order to identify the presence of putative essential genes within the final dataset of 105 sequences, these proteins were queried against the Eukaryote Database of Essential Genes using JACKHMMER (with an *e*-value of 10^−20^) and 93 associations were found for 23 query sequences. Upon removal of false positives based on query coverage (>75%), 12 true positives were found to have associations to 32 DEG sequences. A phylogenetic tree was constructed to identify closer clustering of these true positives with their corresponding DEG hits. MUSCLE present within MEGA 5.0 was used to align the sequences with 10 rounds of iterations while Maximum likelihood approach with JTT (Jones et al., 1992) model and 100 bootstrap replications were used to build the tree. Fig-tree was used to visualize the tree provided as Figure 8. The unedited tree is shown in Supplementary Figure 7 for further information related to bootstrap values.

**Figure 9:**
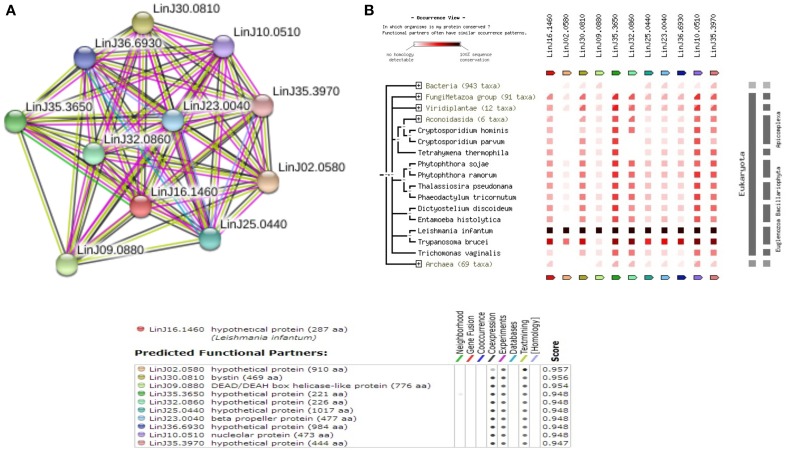
**(A)** COG network of the hypothetical protein (E9BCZ9) and its interacting members. **(B)** Phylogenetic co-occurrence pattern of the protein (E9BCZ9) and its interacting members, seen to be conserved across Eukaryotes.

Among the 12 true positives, 5 sequences belonged to the super family of WD40 repeats suggesting their roles in various protein-protein interactions. A String-DB based gene neighborhood and interaction analysis was performed using the remaining 7 true positives as query. Detailed analysis of E9BCZ9, suggests plausible roles in ribosomal biogenesis in eukaryotes. All of its interacting members are conserved across eukaryotes as displayed in Figures 9A,B. Careful homology based sequence analysis of E9BCZ9 suggests its close association with Nep1 (methyltransferase), a protein that plays roles in the ribosome biogenesis which is conserved across Eukaryotes and Achaea. Nep1 are a class of enzymes that catalyze methylation reaction during the steps of rRNA processing necessary for the generation of 40s ribosomal subunits (Eschrich et al., 2002).

The *Leishmania donovani* counterparts (sharing >90% similarity and coverage) for the *Leishmania infantum* proteins represented in the string network are described in Supplementary Table 7. Pathway association performed via KAAS had also associated E9BCZ9 to the Ribosomal biogenesis pathway (refer Supplementary Table 2). Further, essential gene analysis strongly associates E9BCZ9 to the ribosomal biogenesis. Thus, information obtained from String, DEG and KAAS, collectively increases the confidence of predictions, and in associating E9BCZ9 to Nep1 in the ribosomal biogenesis pathway.

## Conclusion

Effective utilization of the various available bioinformatics tools has enabled the successful characterization of a set of hypothetical proteins within *Leishmania donovani*. Putative functions have been assigned to 105 hypothetical sequences which have a domain spanning more than half of its length and a GO term association. Amongst the 105 sequences, 27 sequences have revealed their associations with a KEGG pathway. Exploiting the information from KEGG and via homology approaches, 4 pathways namely, Ubiquinone biosynthesis, Fatty acid elongation in Mitochondria, Fatty Acid Elongation in ER and Seleno-cysteine Metabolism, have been reconstructed.

Subtractive genomics studies have elicited that ubiquinone pathway is a potential drug target in Mycobacterium *tuberculosis* (Anishetty et al., 2005). Pathways related to Fatty acid and lipid metabolism have been studied and their roles in altering the Miltefosine induced drug resistance in Leishmania *promastigotes* is established (Rakotomanga et al., 2005). These understandings and correlations toward the Leishmania proteome will certainly enable better appreciation of Miltefosine induced drug resistance mechanisms, and aid in the design of better treatment strategies against Leishmaniasis. Furthermore, finer points about 7 essential genes involved in crucial metabolic pathways in *Leishmania donovani* have been derived, which facilitates their exploration as plausible drug targets. Additionally, a new gene cluster related to ribosomal biogenesis also has been elucidated in detail.

In summary, the use of simple, yet robust *insilico* approaches, have been highlighted to prove the immense utilities of the knowledge databases and tools, toward characterization of hypothetical proteins from *Leishmania donovani*, whose genome wide information is elusive due to the presence of huge number of uncharacterized sequences.

### Conflict of interest statement

The authors declare that the research was conducted in the absence of any commercial or financial relationships that could be construed as a potential conflict of interest.

## Supplementary material

Supplementary Figure 1 **The sequence similarity distribution from blast2go**.

Supplementary Figure 2 **The *E*-value distribution of blast2go hits**.

Supplementary Figure 3 **KEGG representation of the ubiquinone biosynthesis pathway**. E9BL43 (part of 105 sequence dataset) is associated to coq7 (highlighted in red) in the pathway.

Supplementary Figure 4 **KEGG representation of the Fatty acid elongation pathway in Mitochondria**. E9B7Z4 (part of 105 sequence dataset) is associated to trans-2-enoyl-CoA reductase—1.3.1.38 (highlighted in red) in the pathway.

Supplementary Figure 5 **KEGG representation of the Fatty Acid elongation in ER**. E9BQF5 (part of 105 sequence dataset) is associated to 3-oxoacyl-coA reductase (highlighted in red) in the pathway.

Supplementary Figure 6 **MetaCyc representation of Seleno-cysteine Metabolism**. E9B9Y6 (part of 105 sequence dataset) is associated to Seleno-cysteine synthase (EC 2.9.1.2) in the pathway.

Supplementary Figure 7 **Unedited Phylogenetic tree with bootstrap values for 12 query sequences associated to 32 DEG hits**.

Supplementary Table 1 **Information containing GO terms, Interproscan domain association, homologs picked during the BLAST step in the BLAST2GO analysis for the 105 sequences**.

Supplementary Table 2 **Sequence wise association of pathway information obtained from KASS for 27 proteins**.

Supplementary Table 3 **Table showing the sequence information in the Ubiquinone biosynthesis pathway along with the sequence information for other members of the genus Leishmania**. LD, *Leishmania donovani;* DGR, *Drosophila grimshavi*.

Supplementary Table 4 **Table showing the sequence information in the fatty acid elongation (Mitochondria) pathway along with the sequence information for other members of the genus Leishmania**. LD, *Leishmania donovani;* DGR*, Drosophila grimshavi.*

Supplementary Table 5 **Table showing the sequence information in the Fatty Acid elongation in ER pathway along with the sequence information for other members of the genus Leishmania**. LD, *Leishmania donovani;* DGR*, Drosophila grimshavi.*

Supplementary Table 6 **Table showing the sequence information in the Seleno-cysteine metabolism pathway along with the sequence information for other members of the genus Leishmania**. LD, *Leishmania donovani;* DGR*, Drosophila grimshavi*.

Supplementary Table 7 **Table showing the corresponding ortholog for each of the protein within *Leishmania donovani***. The String interaction for the E9BCZ9 was performed against *Leishmania infantum* since *L. donovani* is not available in String-DB.
