# Supplementary figures and images for: Insilico analysis of hypothetical proteins unveils putative metabolic pathways and essential genes in Leishmania donovani
Source: Front Genet. 2014 Aug 26;5:291. doi: 10.3389/fgene.2014.00291 (PMC4144268; doi:10.3389/fgene.2014.00291)

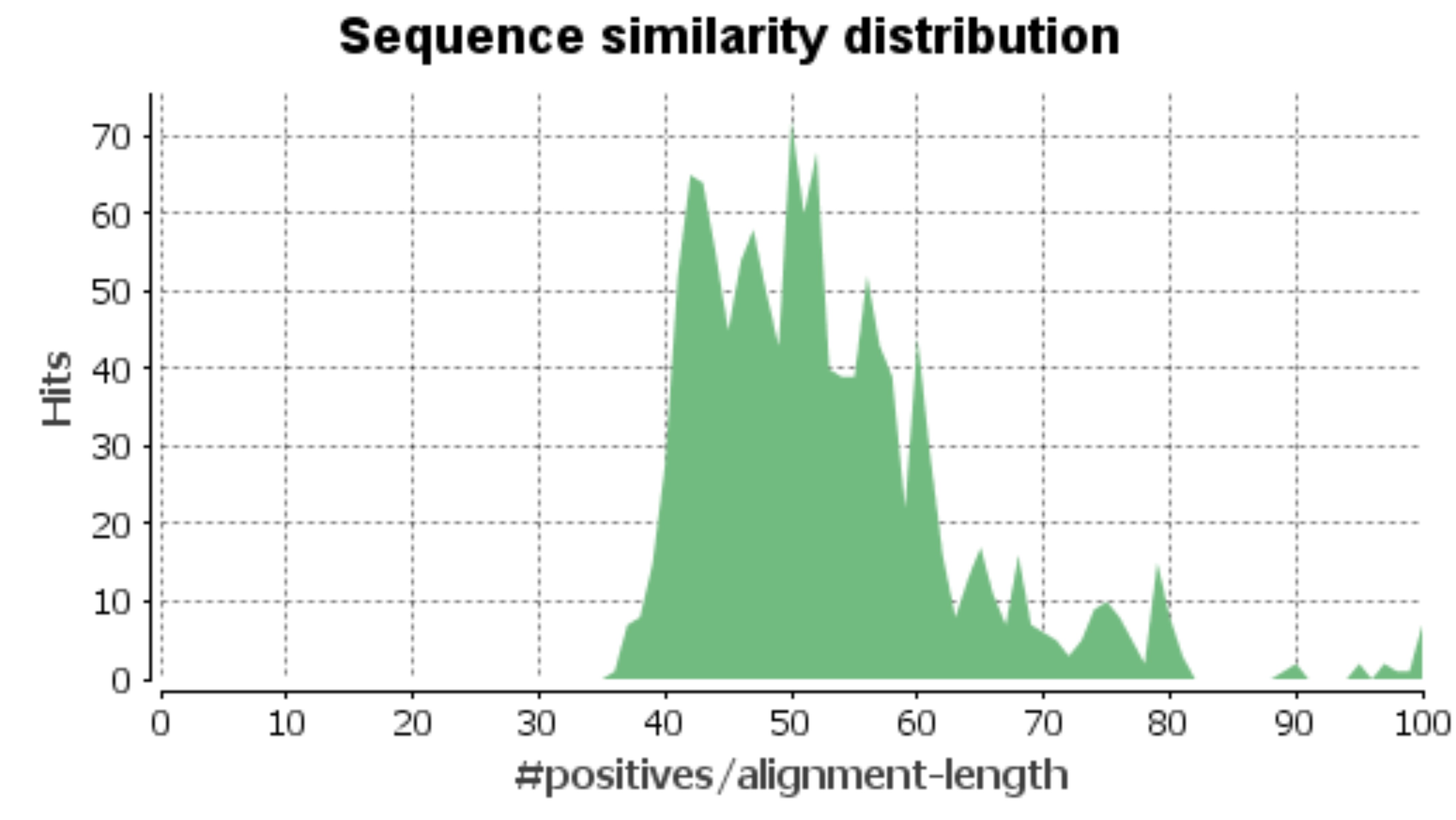

Supplement: Supplementary Figure 1 — The sequence similarity distribution from blast2go. [file Image1.JPEG]

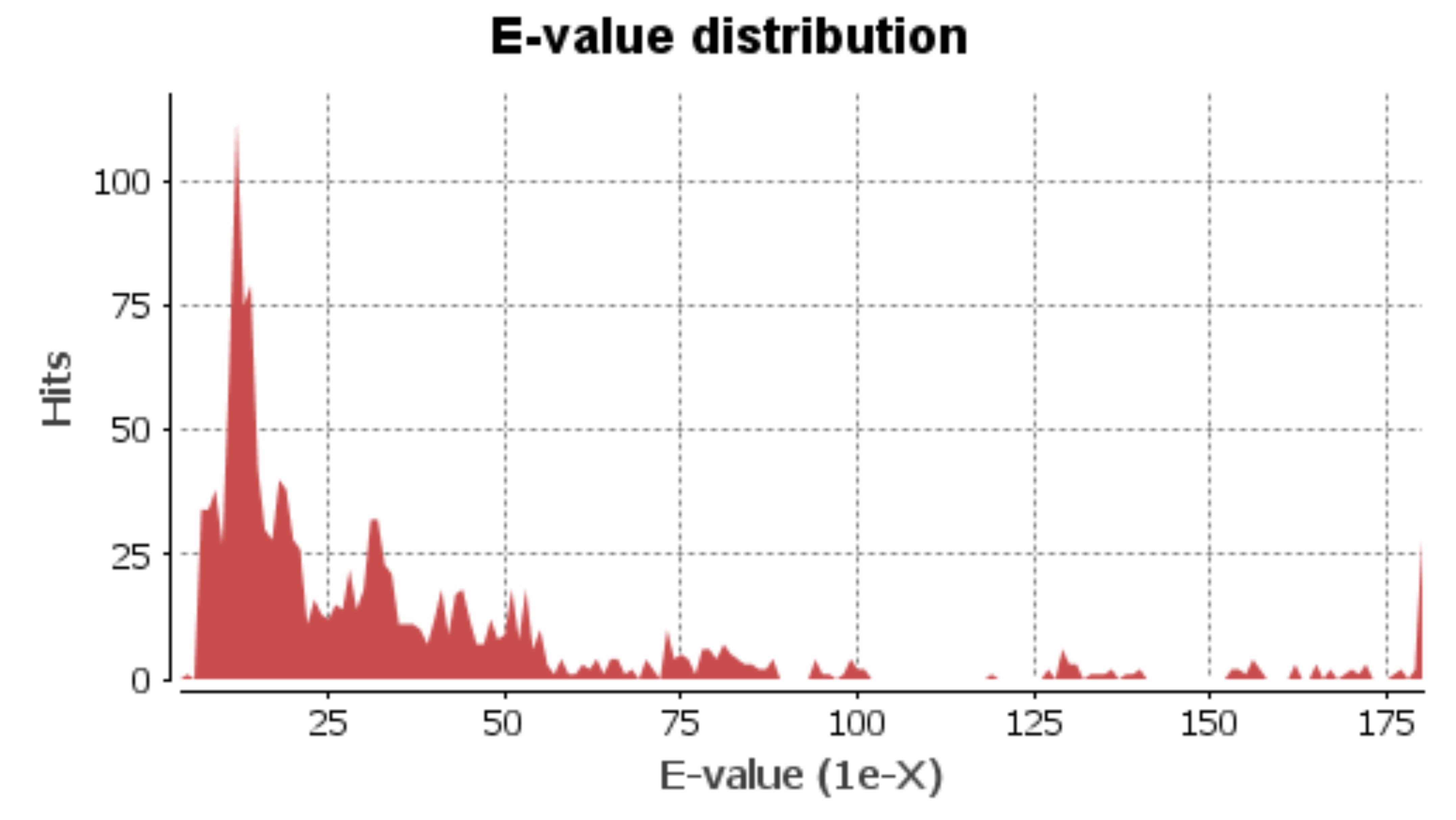

Supplement: Supplementary Figure 2 — The E-value distribution of blast2go hits. [file Image2.JPEG]

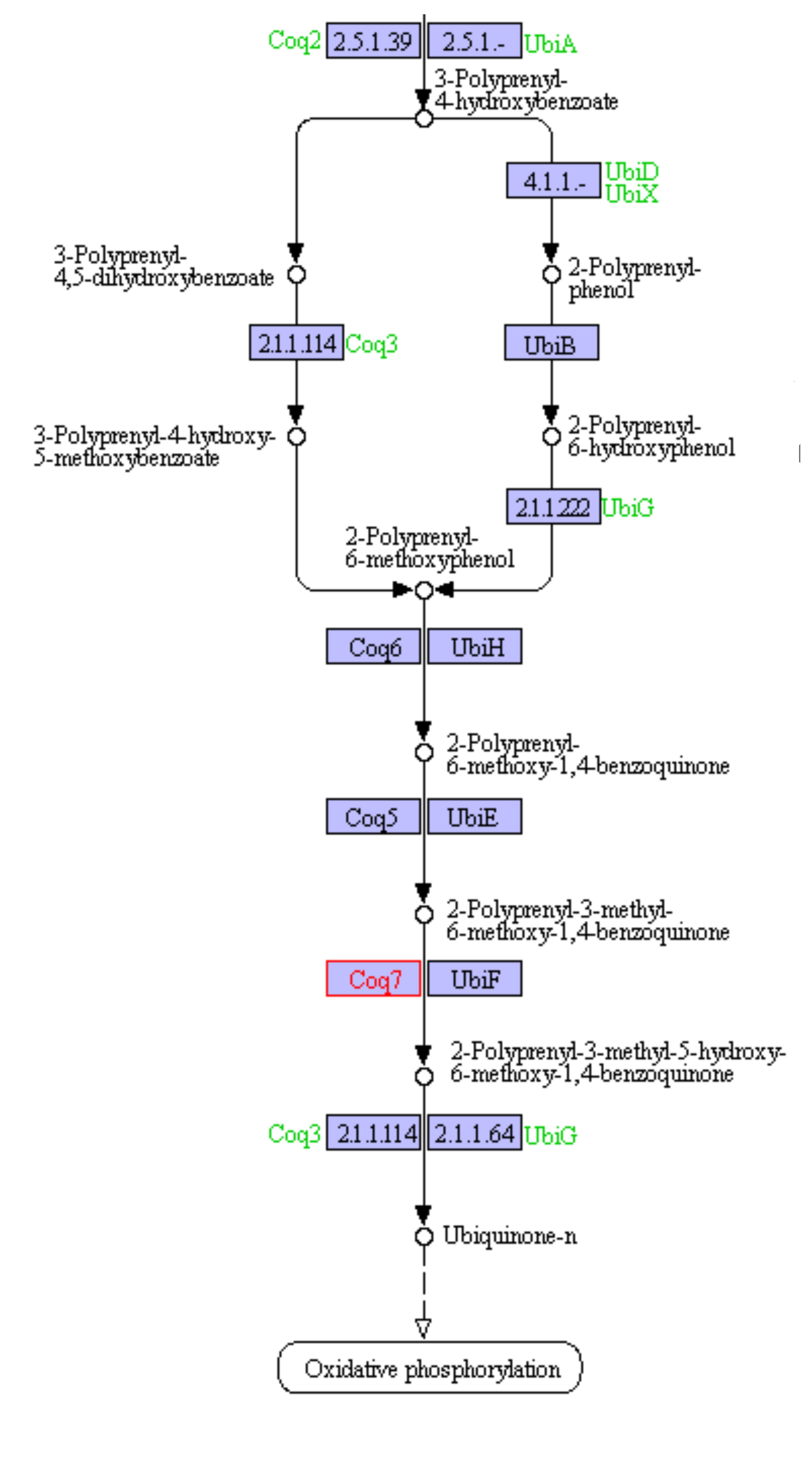

Supplement: Supplementary Figure 3 — KEGG representation of the ubiquinone biosynthesis pathway. E9BL43 (part of 105 sequence dataset) is associated to coq7 (highlighted in red) in the pathway. [file Image3.JPEG]

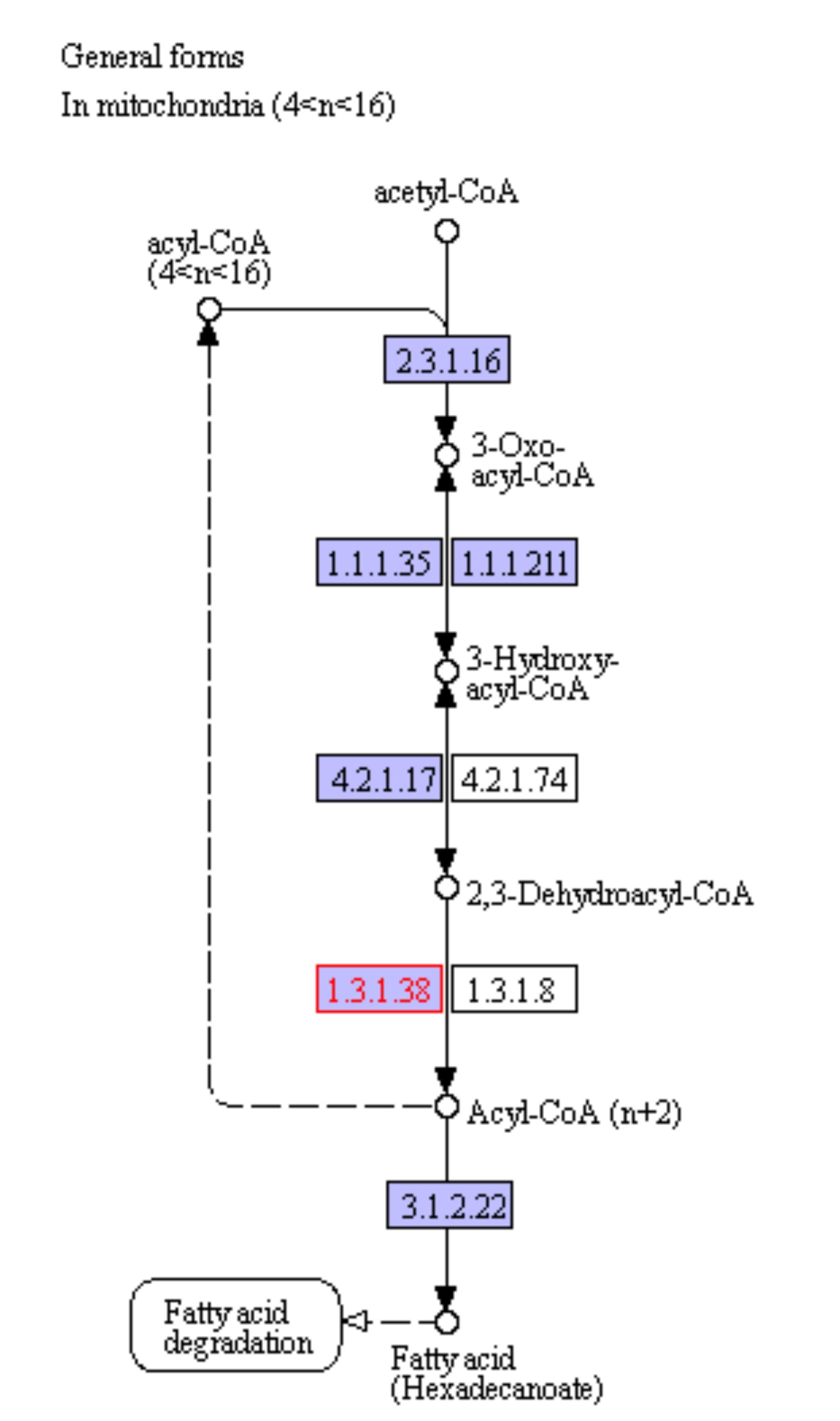

Supplement: Supplementary Figure 4 — KEGG representation of the Fatty acid elongation pathway in Mitochondria. E9B7Z4 (part of 105 sequence dataset) is associated to trans-2-enoyl-CoA reductase—1.3.1.38 (highlighted in red) in the pathway. [file Image4.JPEG]

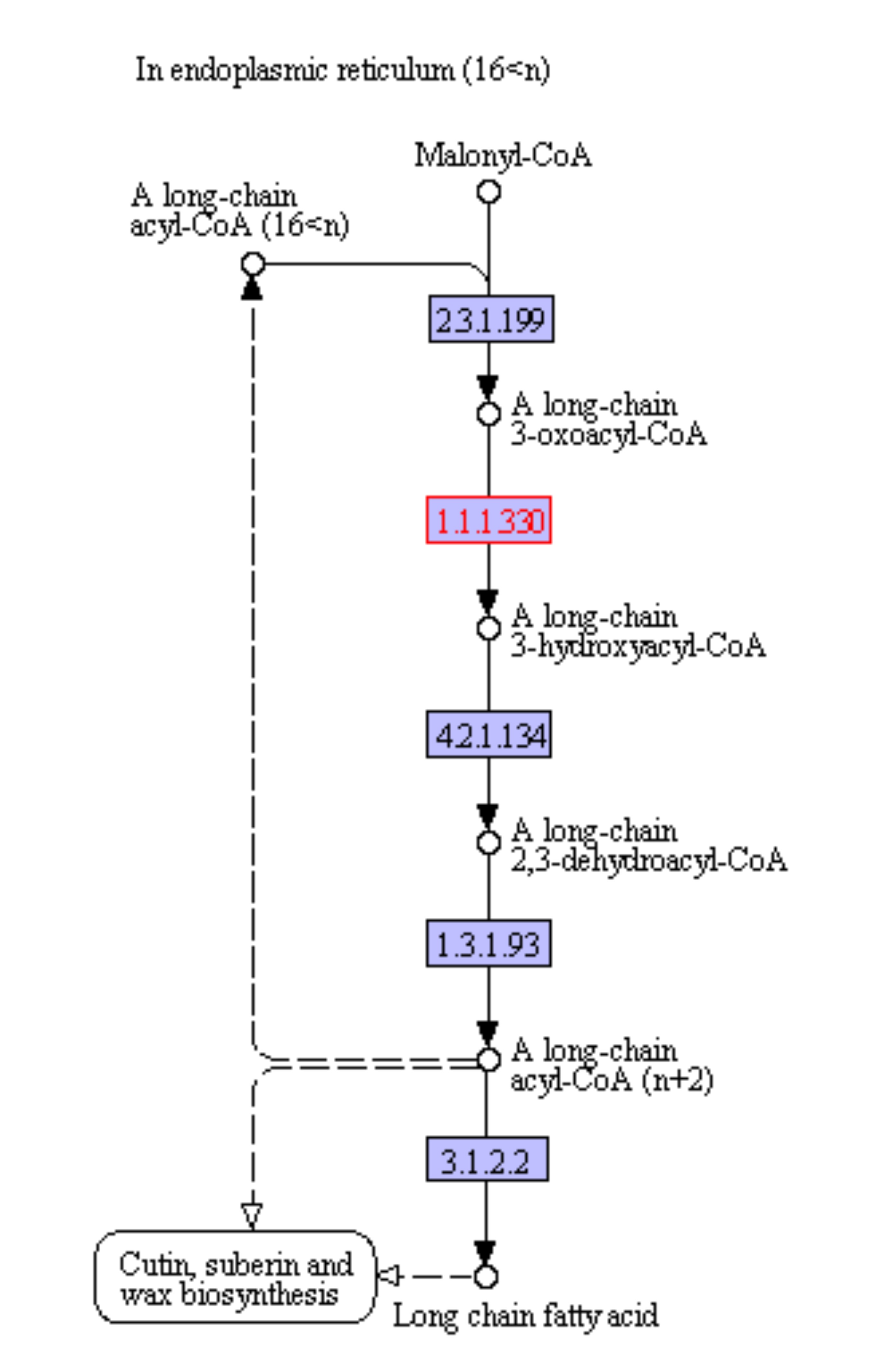

Supplement: Supplementary Figure 5 — KEGG representation of the Fatty Acid elongation in ER. E9BQF5 (part of 105 sequence dataset) is associated to 3-oxoacyl-coA reductase (highlighted in red) in the pathway. [file Image5.JPEG]

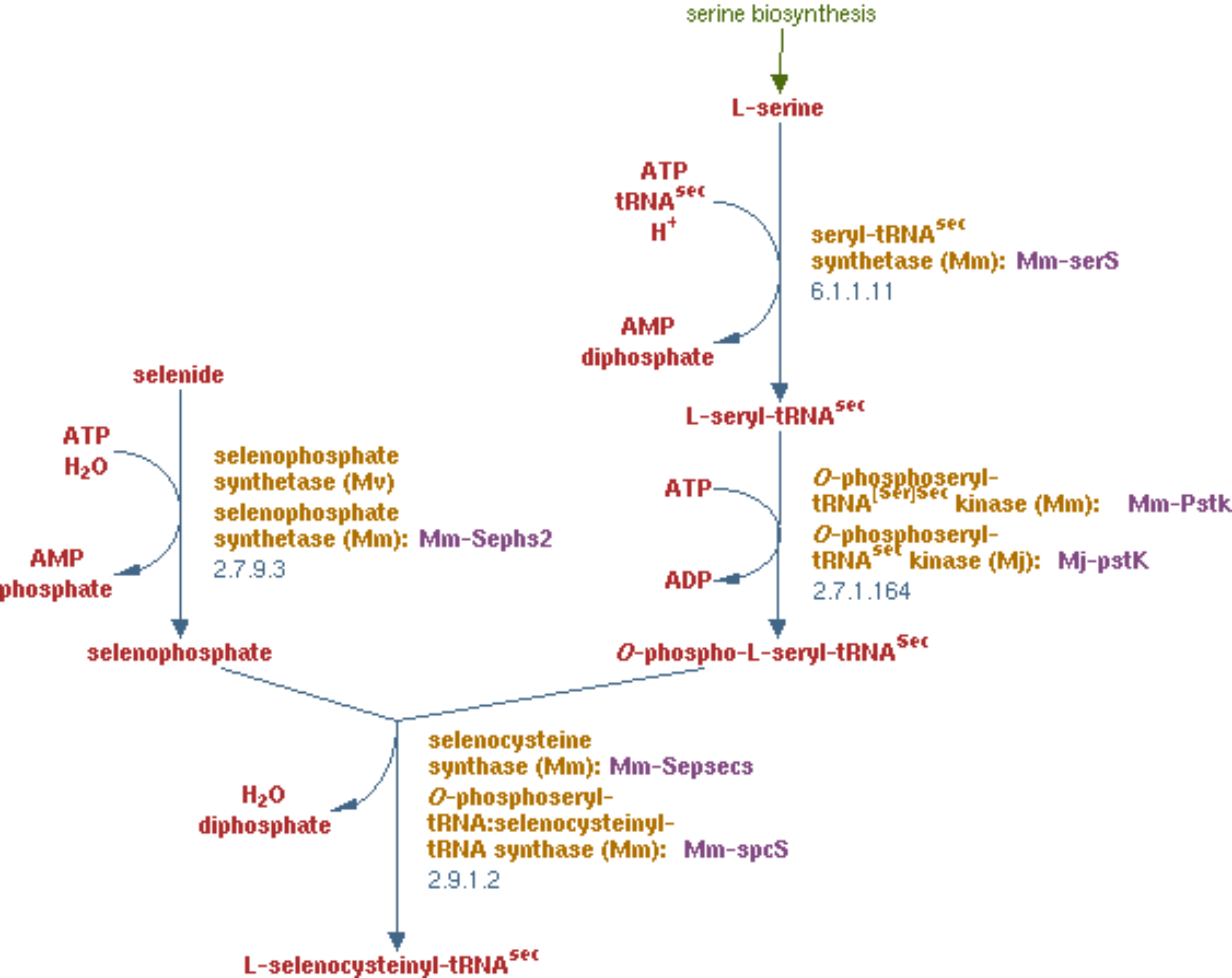

Supplement: Supplementary Figure 6 — MetaCyc representation of Seleno-cysteine Metabolism. E9B9Y6 (part of 105 sequence dataset) is associated to Seleno-cysteine synthase (EC 2.9.1.2) in the pathway. [file Image6.JPEG]

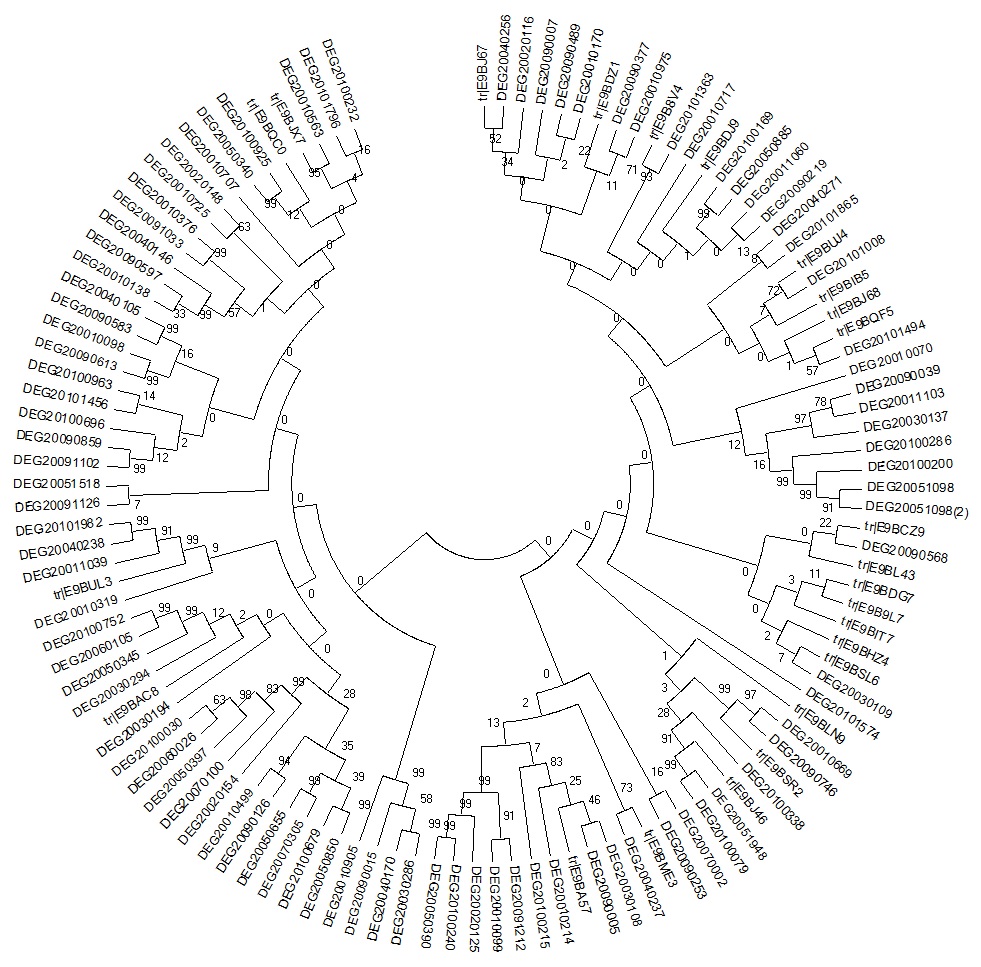

Supplement: Supplementary Figure 7 — Unedited Phylogenetic tree with bootstrap values for 12 query sequences associated to 32 DEG hits. [file Image7.JPEG]
