# Supplementary material for: Insilico analysis of hypothetical proteins unveils putative metabolic pathways and essential genes in Leishmania donovani
Source: Front Genet. 2014 Aug 26;5:291. doi: 10.3389/fgene.2014.00291 (PMC4144268; doi:10.3389/fgene.2014.00291)
Supplement: Supplementary Table 2 — Sequence wise association of pathway information obtained from KASS for 27 proteins. [file Table2.DOCX]

| **QUERY ID** | **KEGG Orthology ID** | **PATHWAY** |
| --- | --- | --- |
| E9BJ46 | K13137 | 03013- RNA TRANSPORT |
| E9BQF5 | K10251 | 01040- BIOSYNTHESIS OF FATTY ACIDS |
| E9BDG7 | K06210 | 00760- NICOTINATE & NICOTINAMIDE METABOLISM |
| E9BSB9 | K01557 | 00350- TYROSINE METABOLISM |
| E9BJR7 | K08964 | 00270- CYSTEINE & METHIONINE METABOLISM |
| E9B7Z4 | K07512 | 00062- FATTY ACID ELONGATION |
| E9BHZ4 | K13346 | 04146-PEROXISOME |
| E9BSW7 | K12197 | 04144- ENDOCYTOSIS |
| E9BJ67 | K12191 | 04144- ENDOCYTOSIS |
| E9BHM4 | K14617 | 04977- VITAMIN DIGESTION AND ABSORPTION |
| E9B9Y6 | K03341 | 00450- SELENO COMPUND METABOLISM |
| E9BCZ9 | K14568 | 03008- RIBOSOME BIOGENESIS IN EUKARYOTES |
| E9BQC0 | K13989 | 04141- PROTEIN PROCESSING IN ENDOPLASMIC RETICULUM |
| E9BJ68 | K14832 | 03008- RIBOSOME BIOGENESIS |
| E9BL43 | K06134 | 00130- UBIQUINONE AND TERPENOID BIOSYNTHESIS |
| E9BI41 | K14397 | 03015- mRNA SURVEILLANCE PATHWAY |
| E9BUJ4 | K02428 | 00230- PURINE METABOLISM  00240- PYRIMIDINE METABOLISM |
| E9BS73 | K03953 | 00190- oxidative Phosphorylation |
| E9BH82 | K01641 | 00072- synthesis & degradation of ketone bodies  00650- butanoate metabolism  00900- Terpenoid backbone synthesis  00280- valine, Leucine & Isoleucine degradation |
| E9BCP7 | K01590 | 00340- Histidine metabolism |
| E9BPV6 | K14406 | 03015- mRNA surveillance pathway |
| E9BJE7 | K14568 | 03008- ribosome biogenesis in eukaryotes |
| E9BKN8 | K11091 | 03040- Histidine metabolism |
| E9BUZ5 | K14168 | 04122- sulphur relay system |
| E9BQA0 | K10251 | 00062- fatty acid elongation  01040- biosynthesis of unsaturated fatty acids  00140- steroid harmone biosynthesis |
| E9BHW3 | K01630 | 00053- Ascorbate and Aldarate metabolism |
| E9BBK3 | K13985 | 04723- Retrograde endocannabinoid signalling |

Supplementary table 2
