# Supplementary material for: Insilico analysis of hypothetical proteins unveils putative metabolic pathways and essential genes in Leishmania donovani
Source: Front Genet. 2014 Aug 26;5:291. doi: 10.3389/fgene.2014.00291 (PMC4144268; doi:10.3389/fgene.2014.00291)
Supplement: Supplementary Table 3 — Table showing the sequence information in the Ubiquinone biosynthesis pathway along with the sequence information for other members of the genus Leishmania. LD, Leishmania donovani; DGR, Drosophila grimshavi. [file Table3.DOCX]

Table S3: Table showing the sequence information in the Ubiquinone biosynthesis pathway along with the sequence information for other members of the genus Leishmania. LD- *Leishmania donovani,* DGR*- Drosophila grimshavi*
