# Supplementary material for: Insilico analysis of hypothetical proteins unveils putative metabolic pathways and essential genes in Leishmania donovani
Source: Front Genet. 2014 Aug 26;5:291. doi: 10.3389/fgene.2014.00291 (PMC4144268; doi:10.3389/fgene.2014.00291)
Supplement: Supplementary Table 5 — Table showing the sequence information in the Fatty Acid elongation in ER pathway along with the sequence information for other members of the genus Leishmania. LD, Leishmania donovani; DGR, Drosophila grimshavi. [file Table5.DOCX]

Table S5: Table showing the sequence information in the Fatty Acid elongation in ER pathway along with the sequence information for other members of the genus Leishmania. LD: *Leishmania donovani,* DGR*- Drosophila grimshavi*
