# Supplementary material for: Insilico analysis of hypothetical proteins unveils putative metabolic pathways and essential genes in Leishmania donovani
Source: Front Genet. 2014 Aug 26;5:291. doi: 10.3389/fgene.2014.00291 (PMC4144268; doi:10.3389/fgene.2014.00291)
Supplement: Supplementary Table 7 — Table showing the corresponding ortholog for each of the protein within Leishmania donovani. The String interaction for the E9BCZ9 was performed against Leishmania infantum since L. donovani is not available in String-DB. [file Table7.DOCX]

| ***Leishmania infantum*** | ***Leishmania donovani*** |
| --- | --- |
| LinJ16.1460- A4HX36 | E9BCZ9 |
| LinJ02.0580- A4HRR1 | E9B7L0 |
| LinJ30.0810- A4I576 | E9BLE3 |
| LinJ09.0880- A4HU04 | E9B9X5 |
| LinJ35.3650- A4IBP5 | E9BSN0 |
| LinJ32.0860- A4I7L3 | E9BNH6 |
| LinJ25.0440- A4I186 | E9BHE8 |
| LinJ23.0040- A4I040 | E9BG24 |
| LinJ36.6930- A4IE52 | E9BUF0 |
| LinJ10.0510- A4HUC9 | E9BA69 |
| LinJ35.3970- A4IB56 | E9BSR2 |

Table S7: Table showing the corresponding ortholog for each of the protein within *Leishmania donovani*. The String interaction for the E9BCZ9 was performed against *Leishmania infantum* since *L. donovani* is not available in String-DB.
